# Prevalence and Outcome of Infections Caused by Staphylococcus aureus Strains Harboring the Panton-Valentine Leukocidin Gene

**DOI:** 10.7759/cureus.81687

**Published:** 2025-04-04

**Authors:** Vrushali H Thakar, Mahadevan Kumar, Meera Modak, Neetu Mehrotra, Deepa Devhare, Aishwarya Babu, Bharati Dalal, Sania Paul, Lata Yadav, Shailaja Sawant

**Affiliations:** 1 Infectious Diseases, Clinical Microbiology, and Antimicrobial Stewardship Programs (AMSP), Bharati Vidyapeeth's Medical College, Pune, IND; 2 Infectious Diseases, Bharati Vidyapeeth's Medical College, Pune, IND

**Keywords:** community-acquired methicillin-resistant staphylococcus aureus (ca-mrsa), hospital-acquired methicillin-resistant staphylococcus aureus (ha-mrsa), methicillin-resistant staphylococcus aureus, pvl gene, scc iv gene

## Abstract

Background: *Staphylococcus aureus*, especially Methicillin-resistant *S. aureus* (MRSA), is responsible for various hospital-acquired (HA) and community-acquired (CA) infections. Mec A gene responsible for methicillin resistance is encoded in Staphylococcal cassette chromosome gene (SCC). CA-MRSA strains often carry SCC mec IV / SCC mec V and Panton-Valentine leukocidin (PVL) genes. PVL-producing MRSA can cause severe skin or soft tissue infections.

Aim: This study estimates the prevalence and outcome of infections caused by PVL-producing *S. aureus* strains in outdoor and indoor patients of a tertiary care hospital in Western Maharashtra, India.

Method: This cross-sectional study included 274 *S. aureus* strains isolated from various clinical samples during the study period. Detection of Methicillin resistance was done by the cefoxitin disk method and by automated antimicrobial susceptibility (Vitek 2) method. Multiplex PCR was done for the detection of MecA, SCC type IV, Nuc, and PVL gene using appropriate primers.

Results: Out of 274 *S. aureus* strains, 151 (55%) were methicillin resistant. The PVL gene was detected in 187 (70.8%) strains and SCC IV in 116 (43.9%) strains. Mec A was detected in all MRSA strains. Both PVL-producing MRSA (83.4%) and MSSA (78.78) strains were isolated commonly from pus samples. Patients with PVL-producing *S. aureus *infections required more surgical interventions (18.1%) as compared to those with PVL-negative infections (5.19%) (p-value = 0.006). The PVL gene was associated with SCC IV in 84 (58.7%) strains, while 35 (24.4%) PVL-positive strains were not associated with SCC IV. Few SCC IV and PVL-positive *S. aureus* strains (17 strains) have produced HA infections.

Conclusion: The prevalence of the PVL gene is 70.8% in *S. aureus*. PVL and SCC IV genes are markers of CA infection. MRSA strains harbouring PVL and SCC IV gene producing HA infections is a cause of concern. This suggests infiltration of SCC IV gene producing *S. aureus* strains in hospital settings. Proper antibiotic stewardship practices, strict aseptic techniques, identification, and treatment of carriers are needed to control the spread of CA-MRSA in hospitals and in the community.

## Introduction

Methicillin-resistant *Staphylococcus aureus* (MRSA) is a common cause of hospital-acquired (HA) and community-acquired (CA) infections with worldwide occurrence [1]. The resistance of MRSA to β-lactam antibiotics is due to the presence of a modified penicillin-binding protein 2a (PBP 2a) and mecA gene. MecA gene is located in the staphylococcal cassette chromosome (SCCmec). SCC mec types I, II, and III are associated with HA MRSA strains, while SCC mec type IV/ V are seen in community-associated MRSA (CA-MRSA) strains. [2]. The Panton-Valentine leukocidin (PVL) gene is an important cytotoxin produced by some strains of *S. aureus*. Genes associated with the production of PVL toxin are luk S-PV and luk F-PV [3]. The PVL gene is present in most of the community-associated MRSA isolates and is rarely found in hospital isolates; therefore, it is considered a marker of CA strains [4]. The high virulence of CA-MRSA is associated with the presence of the PVL gene [5]. Although the PVL gene was associated with CA-MRSA strains, HA-MRSA strains carrying PVL genes have also been recently reported in cutaneous and invasive infections [6]. Boan et al. observed that PVL-positive infections are associated with a distinct clinical picture, predominantly pyogenic skin and soft tissue infections often requiring surgery, with a higher proportion of patients who are younger with fewer health-care risk factors. However, these patients responded to clindamycin [7].

Eshwara et al., from Manipal, India, reported the occurrence and prevalence of the PVL gene among *S. aureus *bacteremia (SAB) cases. They have demonstrated a 16% prevalence of the PVL gene in all SAB infections [8]. Jaiswal et al.,from North India, have reported a higher prevalence of the PVL gene in skin and subcutaneous infection, pneumonia, and bloodstream infections [9]. The literature search suggested that there are very few Indian studies with reference to the outcome of PVL toxin-associated *S. aureus* infections, so the present study is undertaken to determine the prevalence and outcome of PVL-producing *S. aureus* infections in indoor and outdoor patients of Bharati Vidyapeeth's Medical College, a tertiary care hospital in Western Maharashtra, India.

This article was presented as an oral paper in Microcon-2024, a national conference of the Indian Association of Medical Microbiologists (IAMM) held in Pune, India.

## Materials and methods

A cross-sectional study was conducted in the Department of Microbiology of Bharati Vidyapeeth's Medical College, a tertiary care hospital in Western Maharashtra, India, from September 2022 to August 2023. Various samples like pus, tissue, wound exudate, and blood culture samples from outdoor (OPD) and indoor (IPD) patients of our hospital with yielding growth of *S. aureus* were included. Urine samples that grew *S. aureus* were excluded from the study. The calculated sample size was 253 *S. aureus *strains [10], but we could isolate 274 strains during the study period. 

The identification of *S. aureus *strains and the detection of methicillin resistance was done by using conventional methods (cefoxitin screen) and by automated ID/AST system (Vitek 2 Biomeurix). Strains were preserved in Trypticase Soy Broth for PCR. As 10 (seven MRSA and three MSSA) slants were lost, multiplex PCR for Nuc, mec A, SCC IV, and PVL gene was done on 264 strains only by using appropriate primers [11]. However, this loss did not affect the findings of the study, as the calculated sample size was only 253. The demographic and clinical details of patients were collected.

HA infection was defined as *S. aureus *isolated from patients after 48 hours of admission, patients having a history of hospitalization/catheterization/dialysis in the past six months or surgical site infection within 30 days of surgery, and implant infection within one year of surgery.

CA infections were defined as *S. aureus *isolated from patients within 48 hours of admission or from OPD patients with no history of hospitalization in the past six months and* S. aureus* strains possessing SCC IV gene [12]

The severity of *S. aureus* infections was determined by the following criteria: need for admission in the critical unit, bacteremia, if any surgical interventions (incision/ drainage, debridement, implant removal, amputation, etc.) required, and development of surgical site/implant infection. The outcome was defined as clinical cure or mortality within 30 days of diagnosis. Follow-up of patients was done for three months to assess the severity and outcome of infection.

Polymerase chain reaction

DNA extraction was done by a column-based DNA extraction kit (HIMEDIA) as per the manufacturer’s instructions. The presence of nuc, mecA, and pvl genes was detected by performing multiplex PCR. Each PCR reaction mixture (25 ul per reaction) contained 1 ul (1 U/ul) of Taq DNA polymerase, 2.5 ul PCR buffer (10X), 1 ul of each primer (10 pmol/ul), 2 ul dNTPs (200 mM each), 2.5 ul (10 ng/ul) of template DNA, and 11 ul PCR-grade water. The initial denaturation was done at 94 °C for two minutes. A total of 33 cycles of amplification were done. Each cycle was comprised of 94 °C for one minute, followed by annealing at 55 °C for 45 seconds, 50 °C for 50 seconds, and 50 °C for 45 seconds for mecA, nuc, and pvl genes, respectively, and a common extension of 72 °C for 45 seconds with a final extension at 72 °C for four minutes [9].

A separate PCR reaction was performed for the SCC IV gene [13]. A total of 30 cycles of amplification were performed. The denaturation step was performed at 94 °C for five minutes. Each amplification cycle consisted of 94 °C for one minute, 50°C for one minute, and 72°C for two minutes, and the final extension step was at 72 °C for 10 minutes [10]. The primer sequence is shown in Table 1 [10,11,13].

**Table 1:** Primer sequence of the MecA, Nuc, and PVL genes of Staphylococcus aureus References: [10,11,13]

Gene	Primer sequence	Amplicon size (bp)
Nuc F	GCGATTGATGGTGATACGGTT	270
Nuc R	AGCCAAGCCTTGACGAACTAAAGC	
Mec A F	GTGAAGATATACCAAGTGATT	147
Mec A R	ATGCGCTATAGATTGAAAGGAT	
PVL F	ATCATTAGGTAAAATGTCTGGACATGATCCA	433
PVL R	GCATCAAGTGTATTGGATAGCAAAAGC	
SCC IV a F	GCCTTATTCGAAGAAACCGC	776
SCCIV a R	TACTCTTCTGAAAAGCGTCG	
SCC IV b F	TCTGGAATTACTTCAGCTGC	493
SCC IV b R	AAACAATATTGCTCTCCCTC	

The controls used were ATCC 43300 for the mecA gene and ATCC 25923 for the nuc gene. For PVL and SCC IV genes, in-house positive strains were used. Controls were confirmed by sequencing (the sequencing report is provided in the Appendix). Sterile distilled water was used as the negative control. The amplicon band size was 433 bp for pvl, 147 bp for mec A, 270 bp for nuc, 776 bp for SCC IV a, and 496 for SCC IV b gene [10].

Statistical analysis

The data analysis was performed using IBM SPSS Statistics for Windows, Version 29.0 (released 2023, IBM Corp., Armonk, NY). Data were summarized using percentages. The association between various patients' clinical details (to determine outcome) and PVL status were studied using the Chi-square test. A p-value below 0.05 was considered statistically significant. Univariate analysis was done by logistic regression (95% confidence interval) for checking confounding risk factors.

Ethical committee approval

Ethical approval was obtained from Bharati Vidyapeeth (Deemed to be University) Medical College Institutional Ethics Committee before performing the study. The approval letter number is BVDUMC/IEC/19.

## Results

A total of 274 *S. aureus* strains were isolated from 6,548 clinical samples received during the study period. A total of 186 strains were isolated from IPD patients, of which 47 strains were considered as HA. Eighty-eight strains were isolated from OPD patients. Table 2 shows the sample-wise distribution of strains.

**Table 2:** Sample-wise distribution of Staphylococcus aureus strains Values are presented as N(%).

Sample	Number
Pus	221 (80.6%)
Blood	41 (14.9%)
Fluid	5 (1.82%)
Sputum	2 (0.7%)
Endotracheal tube aspirates	4 (1.45%)
Bronchoalveolar lavage fluid	1 (0.3%)
Total	274

The maximum isolates (221/80.6%) were from pus. Methicillin resistance was detected in 151 strains (55%), while 123 strains were methicillin-susceptible (44.89%). Table 3 shows the distribution of MSSA and MRSA among patients with various clinical conditions.

**Table 3:** Distribution of methicillin-resistant Staphylococcus aureus (MRSA) and methicillin-susceptible Staphylococcus aureus (MSSA) among various clinical infections Values are presented as N (%).

Clinical infections	MSSA (123)	MRSA (151)	Total (274)
Skin and subcutaneous tissue infections	50 (39.68%)	76 (60.31%)	126 (45.9%)
Bone and joint infections	17 (51.51%)	16 (48.48%)	33 (12%)
Eye infections	1 (25%)	3 (75%)	4 (1.45%)
ENT infections	8 (42.1%)	11 (57.8%)	19 (6.93%)
Obstetrics and gynecology infections	4 (66.6%)	2 (33.3%)	06 (2.1%)
Bacteremia	20 (48.78%)	21(51.21%)	41 (14.96%)
Surgical site infections	6 (50%)	6 (50%)	12 (4.37%)
Respiratory tract infections	3 (42.85%)	4 (57.1%)	7 (2.55%)
Intra-abdominal infections	4 (66.6%)	2 (33.3%)	06 (2.18%)
Details not available	10	10	20 (7.29%)

**Figure 1:**
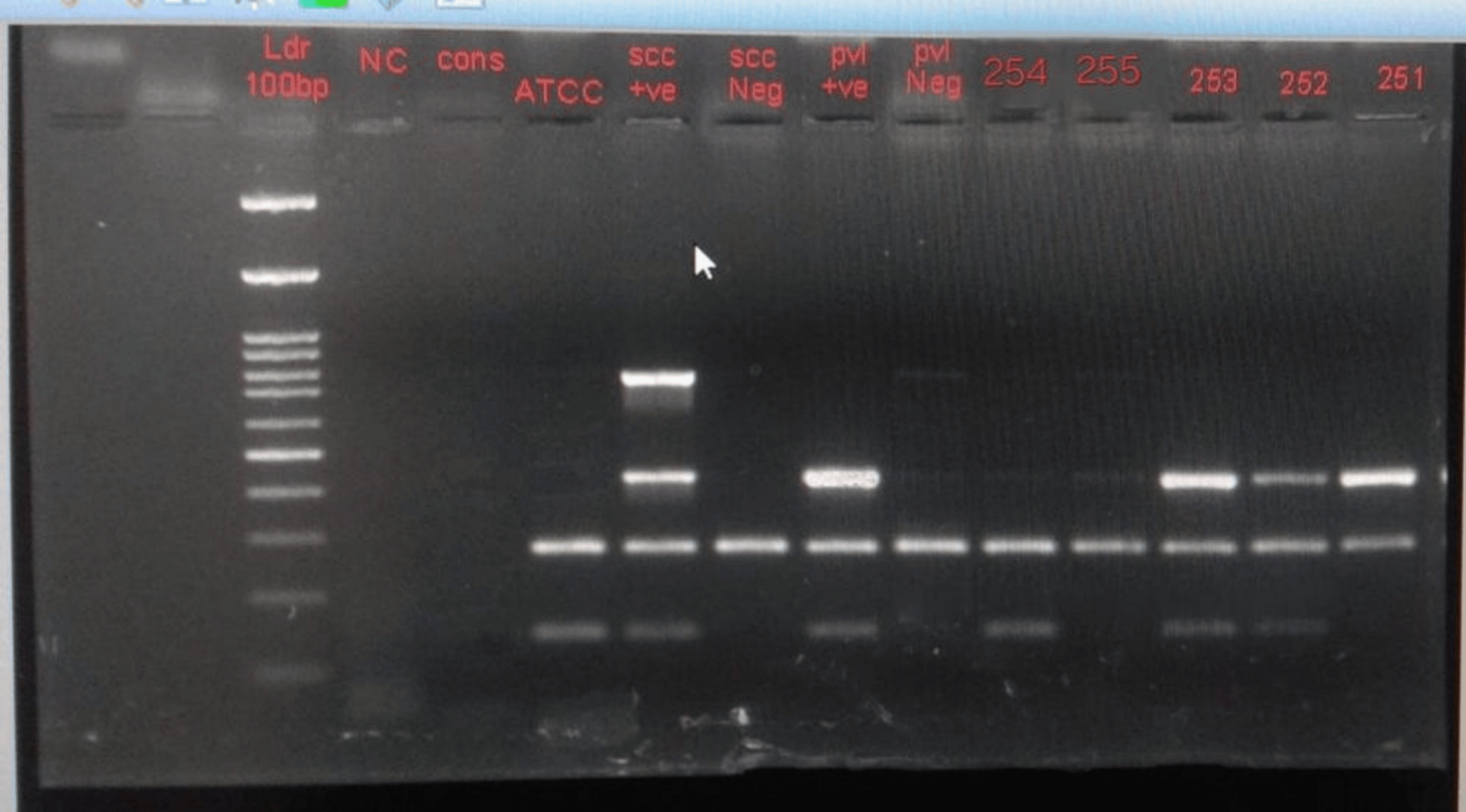
Gel electrophoresis showing the PVL, nuc, SCC IV, and mec A genes Agar gel electrophoresis showing Lane 4 next to DNA ladder -NC,  Lane 5- CONS as negative control for Nuc gene, Lane 6(ATCC MRSA), 7,9 ,11,13,14 -mec positive (147 bp), Lane 7- SCC IV a positive (776bp), Lane 7,9,13,14,15- PVL positive (433 bp).

MRSA and MSSA strains were commonly isolated from skin and subcutaneous infections (126/45.9%). Nuc gene was detected in all *S. aureus* strains. MecA was detected in all MRSA (143-as 7 MRSA slants were lost) strains. PVL gene was detected in 187 (70.8%) *S. aureus* strains and SCC IV gene in 116 (43.9%) strains (Figure 1).

Out of 187 PVL-positive strains, 121 (64.7%) were methicillin-resistant, and 66 (35.29%) were methicillin-susceptible strains. Table 4 shows the distribution of PVL-producing MRSA and MSSA strains among various clinical samples.

**Table 4:** Distribution of Panton-Valentine leukocidin (PVL)-producing methicillin-resistant Staphylococcus aureus (MRSA) and methicillin-susceptible Staphylococcus aureus (MSSA) among various clinical samples Values are presented as N (%).

Clinical sample	PVL-positive MRSA (121)	PVL-positive MSSA (66)
Pus	101 (83.47%)	52 (78.78%)
Blood	14 (11.57%)	12 (18.18%)
Samples from the respiratory tract	5 (4.1%)	2 (3%))
Body fluids	1 (0.8%)	0

Table 5 shows the clinical and demographic details of the patients with PVL-positive and PVL-negative *S. aureus* infections. Multivariate analysis by adjusting the OR to check the age and comorbidity as the confounder did not show any significant risk for PVL-positive *S. aureus* infection.

**Table 5:** Clinical and demographic details of patients having Panton-Valentine leukocidin (PVL)-positive and PVL-negative Staphylococcus aureus infections #-Fisher's exact test was used. Analysis done by logistic regression. *P-value less than 0.05 is considered as significant.

Details of patients	Infected with PVL-positive S. aureus (187)	Infected with PVL- negative S. aureus (77)	Odds ratio (95% confidence interval)	Chi-square value (P-value)
Extremes of age <1 yr or	42 (22.4%)	9 (11.68%)	2.18 (1.00-4.75)	0.04*
Female	70 (37.43%)	23 (29.8%)	1.40 (0.80-2.48)	0.24
Male	117 (62.56%)	54 (70.12%)	0.29 (0.80-2.48)	
Diabetes mellitus	15 (8%)	12 (15.58%)	0.47 (0.21-1.06)	0.07
CKD patients on dialysis	8 (4.27%)	6 (7.79%)	0.53 (0.18-1.58)	0.25
Cancer	3 (1.6%)	5 (6.49%)	0.23 (0.05-1.01)	0.049#*
Hematological disorders	2 (1.06%)	1 (1.29%)	0.82 (0.07-9.20)	0.99#
Previous surgery	22 (11.76%)	11 (14.28%)	0.80 (0.70-1.74)	0.57
Trauma	23 (12.29%)	5 (6.49%)	2.02 (0.74-5.53)	0.16
Hypertensive disorders	10 (5.34%)	12 (15.58%)	0.30 (0.12-0.74)	0.006*
Respiratory complaints	4 (2.13%)	3 (3.89%)	0.54 (0.11-2.45)	0.42#

Patients with PVL-producing *S. aureus* infections required more surgical interventions (18.1%) as compared to those with PVL-negative *S. aureus* infections (5.19%), with p-value of 0.006 (Table 6).

**Table 6:** Outcome of Panton-Valentine leukocidin (PVL) infection Values are presented as N (%). p-value less than 0.05 is considered as significant. *significant. PVL: Panton-Valentine leukocidin, IPD: inpatient department, OPD: outpatient department

	Patients infected with PVL-producing S. aureus (N = 187)	Patients infected with PVL-negative S. aureus (N = 77)	Statistical significance P-value
IPD	112 (59.89%)	44 (57.14%)	0.67
OPD	75 (40.10%)	33 (42.85 %)	0.67
Critical unit admission	33/112 (29.46%)	14(31.81%)	0.77
Mortality	2 deaths	2 deaths	0.32
S. aureus bacteremia	26 (13.9%)	11(14.28%)	0.95
Surgical intervention required	34(18.1%)	4(5.19%)	0.006*
Implant infection (TKR)	3 cases (1.6)	3 cases (3.8%)	0.25
Catheter-related blood stream infection	4 cases (2.1)	5 cases (6.49%)	0.07
Surgical site infection	13 cases (6.9%)	5 cases (6.49%)	0.89

Table 7 shows the association of PVL and SCC IV genes in MRSA strains.

**Table 7:** Association of the Panton-Valentine leukocidin (PVL) and Staphylococcal cassette chromosome gene (SCC) IV genes Values are presented as N (%).

SCC IV	PVL	Number of strains
Positive	Positive	84 (58.7%)
Positive	Negative	4 (2.7%)
Negative	Positive	35 (24.4%)
Negative	Negative	20 (13.9%)

**Figure 2:**
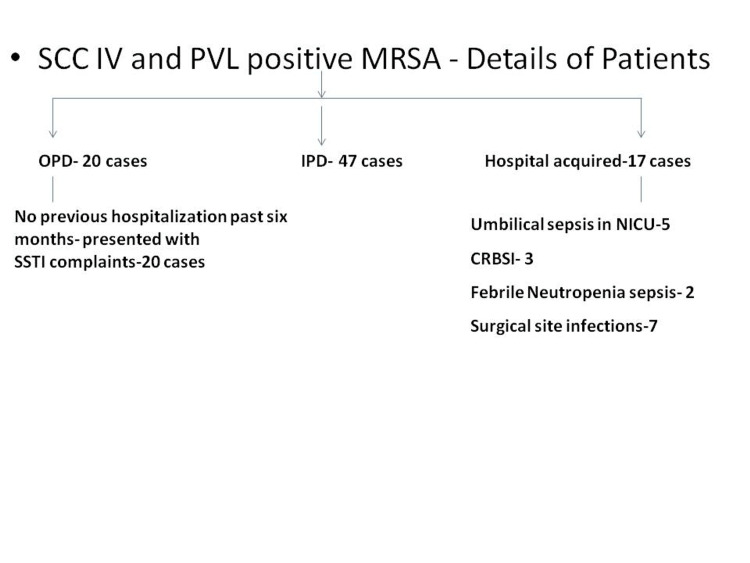
Clinical details of patients having Panton-Valentine leukocidin- and Staphylococcal cassette chromosome gene (SCC) IV-positive methicillin-resistant Staphylococcus aureus (MRSA) infection

MRSA strains were found to be 100% susceptible to daptomycin, teicoplanin, vancomycin, linezolid, rifampicin, and tigecycline. Although the SCC IV gene is seen in CA-MRSA strains, our study has found its presence in hospital strains also (Figure 2)

## Discussion

*S. aureus* is the most common gram-positive organism causing skin and soft tissue infections (SSTI) in the community and invasive infections in hospitalized patients [14]. The rise in the prevalence of MRSA worldwide is alarming as its resistance to antibiotics makes treatment difficult and expensive. Earlier, infections from MRSA were mainly reported from hospital settings, but now there is an emerging trend of these infections in the community also [15,16]. PVL-producing MRSA is less prevalent than PVL-producing MSSA, but it is associated with increased mortality and morbidity in invasive infections [17].

The prevalence of MRSA was 55% in our study. Patil et al. performed a systematic review and meta-analysis to find out the pooled prevalence of MRSA in India. Their study revealed that the pooled prevalence of MRSA varied between 31% and 39% from 2015 to 2019, which increased to 69% in 2020 [18]. Swathirajan et al. reported an alarming upward trend in the prevalence of MRSA infections in HIV patients from 51.8 % in 2012 to 76.6% in 2016 [19].

In this study, MRSA strains were commonly isolated from pus samples (80%), followed by blood (14.9%) and body fluids (1.8%). Adhikari et al. from Nepal also reported maximum isolation of MRSA strains from pus samples (18.5%) [20]. MRSA strains were susceptible to daptomycin, teicoplanin, vancomycin, linezolid, rifampicin, and tigecycline (100% each). Similar findings were observed by Al Zoubi et al. in Iran [21]. Over-the-counter sale of antibiotics and prescription of antibiotics "without indication of infection" by general practitioners are probable factors for developing resistance to commonly used antibiotics [22].

PVL-producing MRSA (83.4%) and MSSA (78.7%) strains were commonly isolated from pus samples. Similar findings were observed by Shohyab et al. in Egypt [23]. PVL gene was detected in 70.8% of *S. aureus* strains in the present study, of which 64.7% strains were methicillin-resistant. In a study conducted by Dhawan et al., 65.8% of SCC mec IV-positive *S. aureus* isolates carried the PVL gene compared to only 26.7% of SCC mec V [24]. D’souza Net al. observed that 64% of MRSA strains produced the PVL gene [25]. In a study conducted by Darboe et al., the PVL gene was detected in 61.4% *of S. aureus* strains isolated from invasive bacteraemia and SSTI cases. PVL prevalence was high in both bacteraemia and SSTI cases from an urban Gambian hospital [10]. The prevalence of the PVL gene was higher in our study than in other studies. This may be due to regional strain differences as the prevalence of PVL varies between various geographical regions.

In the present study, patients with PVL-producing *S. aureus *infections required more surgical interventions (18.1%) as compared to those with PVL-negative *S. aureus *infections (5.19%) (P-value = 0.006). Patients with PVL-positive *S. aureus* infections belonged to extremes of age (22.4%) as compared to those with PVL-negative infections (11.68%) (P-value = 0.04). This could also be a contributing factor in producing severe infections. In a study conducted by Nakaminami et al., it was observed that PVL-positive MRSA were associated with deep-seated skin and subcutaneous infections [26]. Although the PVL gene produced more morbidity, mortality did not differ significantly between PVL-positive and PVL-negative *S. aureus *infections in our study. Grebe et al. found a high level of neutralizing anti-PVL antibodies in Africans [27]. These antibodies may have a role in recovery. In a multinational trial conducted by Baeet al.,it was observed that the PVL gene is commonly associated with CA SSTI, and it is not the only determinant of a patient's outcome. In fact, they have observed a better outcome in PVL-producing MRSA infections than in PVL-negative infections [28]. Dumitrescu O et al. observed the inhibitory effect of clindamycin and linezolid on PVL production. Thus, such infections can be treated with clindamycin [29].

PVL and SCC IV genes are markers of CA *S. aureus* infections. Asghar detected the presence of both SCC mec IV and PVL in the genomic sequence of a CA-MRSA isolate. He found a statistically significant correlation between PVL and SCC mec type IV gene (P value: <0.05) [30]. The PVL genes were initially associated with CA-MRSA isolates and were rarely found in HA MRSA (HA-MRSA) isolates. Later, HA-MRSA isolates carrying the PVL gene became prevalent [31]. Shohayeb M et al. found a higher incidence of PVL-MRSA in HA and CA infections and in strains isolated from HCWs [23]. Factors such as the overuse of antibiotics, prescribing antibiotics for viral infections, and lack of rapid and accurate methods for identification of carriers are responsible for the spread of PVL-producing MRSA in hospitals [24]. SCC IV and V genes are seen in CA-MRSA strains, of which SCC IV is more common [32,33]. Epidemiological definitions of CA-MRSA based on the timing of isolation may result in over estimation of the true prevalence of CA-MRSA as colonization with *S. aureus* may remain for years [12]. Thus, we have used the presence of the SCC IV gene as a marker of CA-MRSA. In the present study, the PVL gene was associated with the SCC IV gene in 84 (58.7%) strains. A total of 35 (24.4%) strains were PVL-positive but SCC IV-negative.

The clinical and demographic details of patients infected with SCC IV- and PVL-positive MRSA (84 cases) were studied. We found that few of these strains produced HA infections like post-operative surgical infections (seven), CRBSI (three), sepsis in febrile neutropenia cases (two), and neonatal umbilical sepsis (five). HA infections caused by CA-MRSA strains have been reported by few authors. This may indicate the spread of such strains in healthcare settings. CA-MRSA may replace traditional HA-MRSA strains in the future [8,28,32,33].

Limitations of the study

We could not detect other SCC genes (types I, II, II, and V) for the epidemiological typing of *S. aureus* strains. Thus, we could not conclude anything about the epidemiology of *S. aureus* strains. Moreover, further sequencing of probable CA-MRSA strains causing HA infections could have been done. The study was performed in a single center. Large multicentric studies will be required before generalizing the findings.

## Conclusions

The prevalence of MRSA was 55% in our study. The prevalence of the PVL gene in *S. aureus* was 70.8%. The PVL gene was common in pus samples. Patients with PVL-producing *S. aureus* infections required more surgical intervention (18.1%) as compared to those with PVL-negative *S. aureus* infections (5.19%). PVL and SCC IV genes are markers of CA infections. MRSA strains harbouring PVL and SCC IV gene producing HA infections is a cause of concern. This suggests infiltration of the SCC IV gene producing MRSA in hospital settings. Proper antibiotic stewardship practices, strict aseptic techniques, and identification and treatment of carriers are needed to control the spread of CA-MRSA in hospitals and in the community.

## Figures and Tables

**Figure 3:**
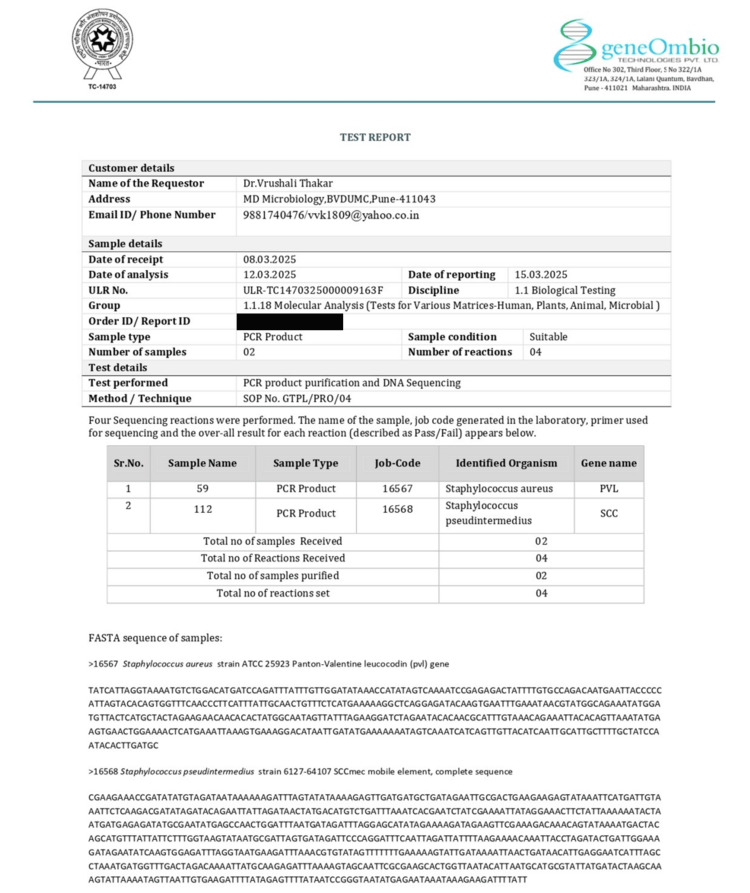
Sequencing result of the Panton-Valentine leukocidin (PVL) and Staphylococcal cassette chromosome gene (SCC) IV genes
